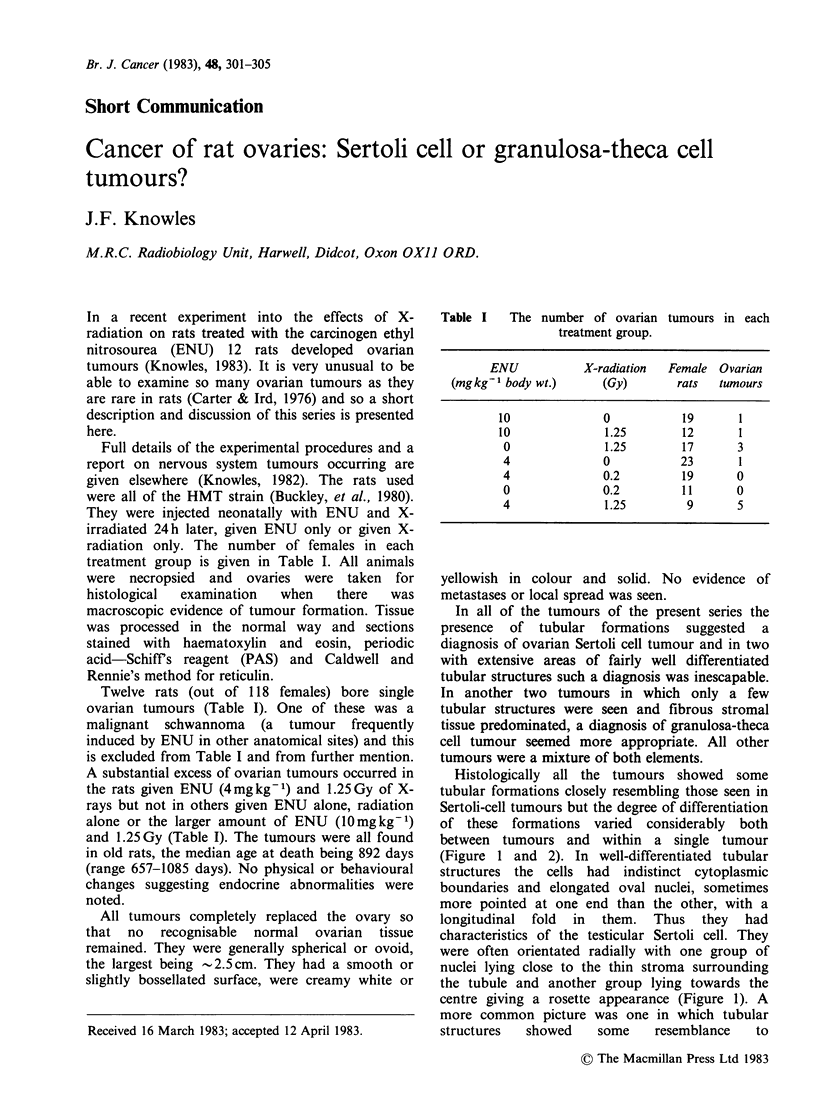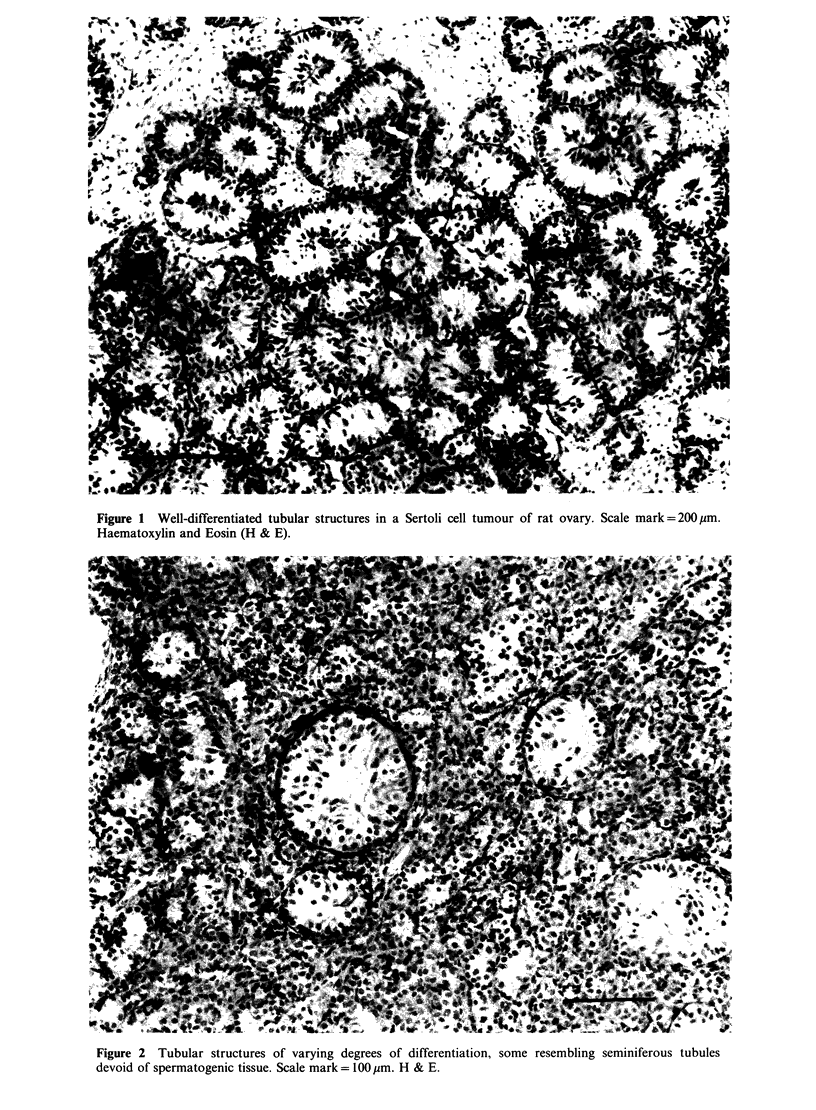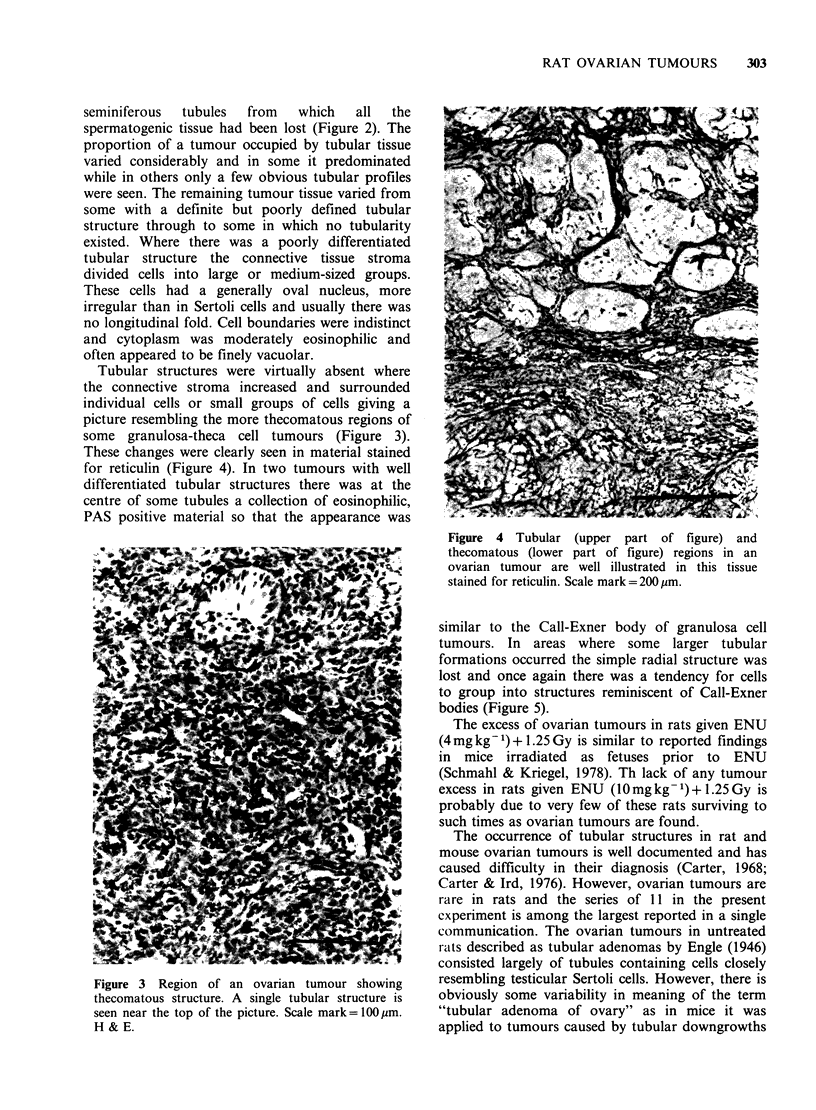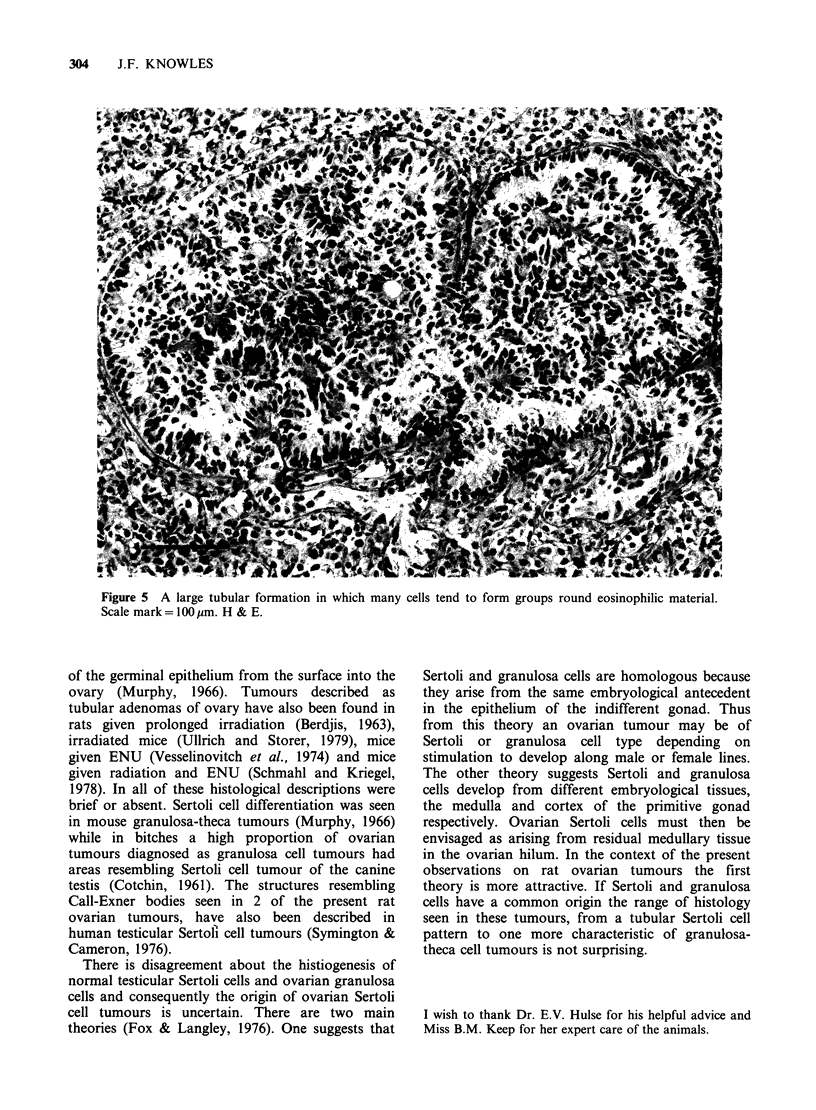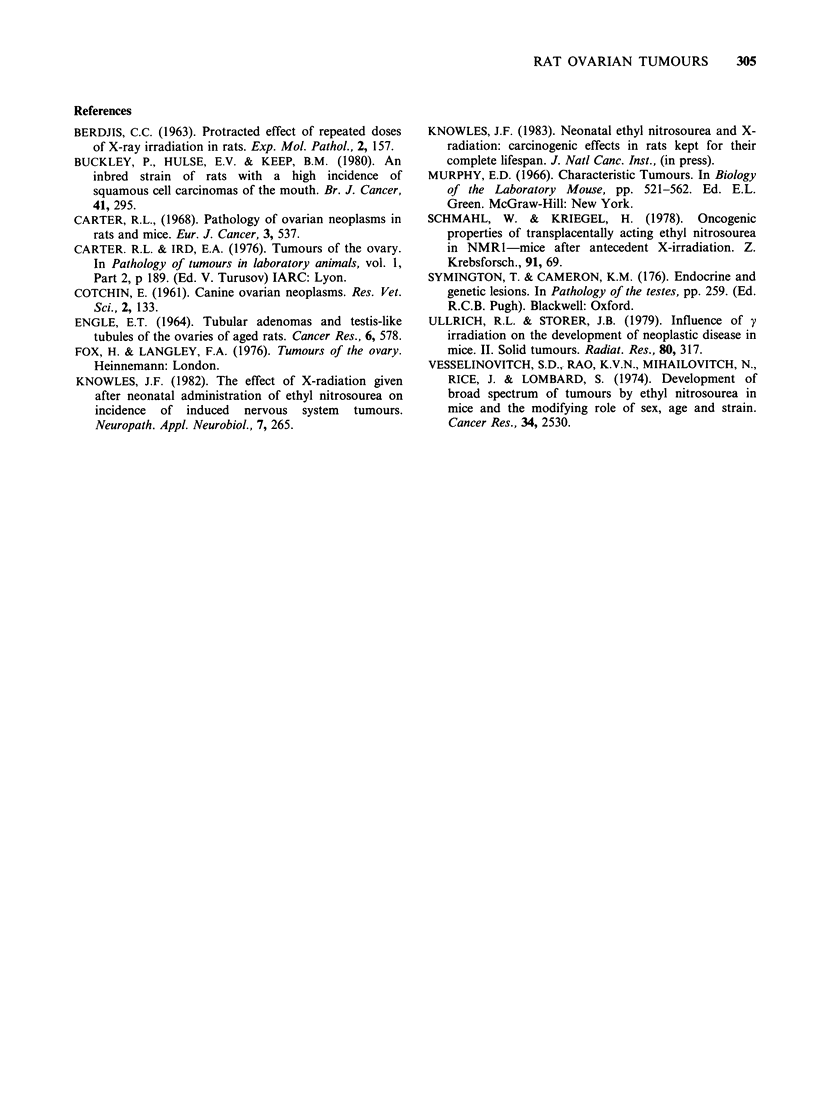# Cancer of rat ovaries: Sertoli cell or granulosa-theca cell tumours?

**DOI:** 10.1038/bjc.1983.185

**Published:** 1983-08

**Authors:** J. F. Knowles

## Abstract

**Images:**


*Br. J. Cancer* (1983), **48**, 301–305

### Short Communication

### Cancer of rat ovaries: Sertoli cell or granulosa-theca cell tumours?

J.F. Knowles

*M.R.C. Radiobiology Unit, Harwell, Didcot, Oxon OX11 ORD.*

In a recent experiment into the effects of X-radiation on rats treated with the carcinogen ethyl nitrosourea (ENU) 12 rats developed ovarian tumours (Knowles, 1983). It is very unusual to be able to examine so many ovarian tumours as they are rare in rats (Carter & Ird, 1976) and so a short description and discussion of this series is presented here.

Full details of the experimental procedures and a report on nervous system tumours occurring are given elsewhere (Knowles, 1982). The rats used were all of the HMT strain (Buckley, *et al.*, 1980). They were injected neonatally with ENU and X-irradiated 24 h later, given ENU only or given X-radiation only. The number of females in each treatment group is given in Table I. All animals were necropsied and ovaries were taken for histological examination when there was macroscopic evidence of tumour formation. Tissue was processed in the normal way and sections stained with haematoxylin and eosin, periodic acid—Schiff's reagent (PAS) and Caldwell and Rennie's method for reticulin.

Twelve rats (out of 118 females) bore single ovarian tumours (Table I). One of these was a malignant schwannoma (a tumour frequently induced by ENU in other anatomical sites) and this is excluded from Table I and from further mention. A substantial excess of ovarian tumours occurred in the rats given ENU (4 mg kg$^{-1}$) and 1.25 Gy of X-rays but not in others given ENU alone, radiation alone or the larger amount of ENU (10 mg kg$^{-1}$) and 1.25 Gy (Table I). The tumours were all found in old rats, the median age at death being 892 days (range 657–1085 days). No physical or behavioural changes suggesting endocrine abnormalities were noted.

All tumours completely replaced the ovary so that no recognisable normal ovarian tissue remained. They were generally spherical or ovoid, the largest being ~2.5 cm. They had a smooth or slightly bossellated surface, were creamy white or yellowish in colour and solid. No evidence of metastases or local spread was seen.

**Table I** The number of ovarian tumours in each treatment group.

| *ENU* (*mg kg*$^{-1}$ *body wt.*) | *X-radiation* (*Gy*) | *Female rats* | *Ovarian tumours* |
|---|---|---|---|
| 10 | 0 | 19 | 1 |
| 10 | 1.25 | 12 | 1 |
| 0 | 1.25 | 17 | 3 |
| 4 | 0 | 23 | 1 |
| 4 | 0.2 | 19 | 0 |
| 0 | 0.2 | 11 | 0 |
| 4 | 1.25 | 9 | 5 |

In all of the tumours of the present series the presence of tubular formations suggested a diagnosis of ovarian Sertoli cell tumour and in two with extensive areas of fairly well differentiated tubular structures such a diagnosis was inescapable. In another two tumours in which only a few tubular structures were seen and fibrous stromal tissue predominated, a diagnosis of granulosa-theca cell tumour seemed more appropriate. All other tumours were a mixture of both elements.

Histologically all the tumours showed some tubular formations closely resembling those seen in Sertoli-cell tumours but the degree of differentiation of these formations varied considerably both between tumours and within a single tumour (Figure 1 and 2). In well-differentiated tubular structures the cells had indistinct cytoplasmic boundaries and elongated oval nuclei, sometimes more pointed at one end than the other, with a longitudinal fold in them. Thus they had characteristics of the testicular Sertoli cell. They were often orientated radially with one group of nuclei lying close to the thin stroma surrounding the tubule and another group lying towards the centre giving a rosette appearance (Figure 1). A more common picture was one in which tubular structures showed some resemblance to

Received 16 March 1983; accepted 12 April 1983.

© The Macmillan Press Ltd 1983

**Figure 1** Well-differentiated tubular structures in a Sertoli cell tumour of rat ovary. Scale mark = 200 $\mu$m. Haematoxylin and Eosin (H & E).

**Figure 2** Tubular structures of varying degrees of differentiation, some resembling seminiferous tubules devoid of spermatogenic tissue. Scale mark = 100 $\mu$m. H & E.

seminiferous tubules from which all the spermatogenic tissue had been lost (Figure 2). The proportion of a tumour occupied by tubular tissue varied considerably and in some it predominated while in others only a few obvious tubular profiles were seen. The remaining tumour tissue varied from some with a definite but poorly defined tubular structure through to some in which no tubularity existed. Where there was a poorly differentiated tubular structure the connective tissue stroma divided cells into large or medium-sized groups. These cells had a generally oval nucleus, more irregular than in Sertoli cells and usually there was no longitudinal fold. Cell boundaries were indistinct and cytoplasm was moderately eosinophilic and often appeared to be finely vacuolar.

Tubular structures were virtually absent where the connective stroma increased and surrounded individual cells or small groups of cells giving a picture resembling the more thecomatous regions of some granulosa-theca cell tumours (Figure 3). These changes were clearly seen in material stained for reticulin (Figure 4). In two tumours with well differentiated tubular structures there was at the centre of some tubules a collection of eosinophilic, PAS positive material so that the appearance was

**Figure 3** Region of an ovarian tumour showing thecomatous structure. A single tubular structure is seen near the top of the picture. Scale mark = 100 μm. H & E.

**Figure 4** Tubular (upper part of figure) and thecomatous (lower part of figure) regions in an ovarian tumour are well illustrated in this tissue stained for reticulin. Scale mark = 200 μm.

similar to the Call-Exner body of granulosa cell tumours. In areas where some larger tubular formations occurred the simple radial structure was lost and once again there was a tendency for cells to group into structures reminiscent of Call-Exner bodies (Figure 5).

The excess of ovarian tumours in rats given ENU ($4\,mg\,kg^{-1}$) + 1.25 Gy is similar to reported findings in mice irradiated as fetuses prior to ENU (Schmahl & Kriegel, 1978). Th lack of any tumour excess in rats given ENU ($10\,mg\,kg^{-1}$) + 1.25 Gy is probably due to very few of these rats surviving to such times as ovarian tumours are found.

The occurrence of tubular structures in rat and mouse ovarian tumours is well documented and has caused difficulty in their diagnosis (Carter, 1968; Carter & Ird, 1976). However, ovarian tumours are rare in rats and the series of 11 in the present experiment is among the largest reported in a single communication. The ovarian tumours in untreated rats described as tubular adenomas by Engle (1946) consisted largely of tubules containing cells closely resembling testicular Sertoli cells. However, there is obviously some variability in meaning of the term "tubular adenoma of ovary" as in mice it was applied to tumours caused by tubular downgrowths

**Figure 5** A large tubular formation in which many cells tend to form groups round eosinophilic material. Scale mark = 100 μm. H & E.

of the germinal epithelium from the surface into the ovary (Murphy, 1966). Tumours described as tubular adenomas of ovary have also been found in rats given prolonged irradiation (Berdjis, 1963), irradiated mice (Ullrich and Storer, 1979), mice given ENU (Vesselinovitch *et al.,* 1974) and mice given radiation and ENU (Schmahl and Kriegel, 1978). In all of these histological descriptions were brief or absent. Sertoli cell differentiation was seen in mouse granulosa-theca tumours (Murphy, 1966) while in bitches a high proportion of ovarian tumours diagnosed as granulosa cell tumours had areas resembling Sertoli cell tumour of the canine testis (Cotchin, 1961). The structures resembling Call-Exner bodies seen in 2 of the present rat ovarian tumours, have also been described in human testicular Sertoli cell tumours (Symington & Cameron, 1976).

There is disagreement about the histiogenesis of normal testicular Sertoli cells and ovarian granulosa cells and consequently the origin of ovarian Sertoli cell tumours is uncertain. There are two main theories (Fox & Langley, 1976). One suggests that Sertoli and granulosa cells are homologous because they arise from the same embryological antecedent in the epithelium of the indifferent gonad. Thus from this theory an ovarian tumour may be of Sertoli or granulosa cell type depending on stimulation to develop along male or female lines. The other theory suggests Sertoli and granulosa cells develop from different embryological tissues, the medulla and cortex of the primitive gonad respectively. Ovarian Sertoli cells must then be envisaged as arising from residual medullary tissue in the ovarian hilum. In the context of the present observations on rat ovarian tumours the first theory is more attractive. If Sertoli and granulosa cells have a common origin the range of histology seen in these tumours, from a tubular Sertoli cell pattern to one more characteristic of granulosa-theca cell tumours is not surprising.

I wish to thank Dr. E.V. Hulse for his helpful advice and Miss B.M. Keep for her expert care of the animals.

### References

BERDJIS, C.C. (1963). Protracted effect of repeated doses of X-ray irradiation in rats. *Exp. Mol. Pathol.*, **2**, 157.

BUCKLEY, P., HULSE, E.V. & KEEP, B.M. (1980). An inbred strain of rats with a high incidence of squamous cell carcinomas of the mouth. *Br. J. Cancer*, **41**, 295.

CARTER, R.L., (1968). Pathology of ovarian neoplasms in rats and mice. *Eur. J. Cancer*, **3**, 537.

CARTER. R.L. & IRD, E.A. (1976). Tumours of the ovary. In *Pathology of tumours in laboratory animals*, vol. 1, Part 2, p 189. (Ed. V. Turusov) IARC: Lyon.

COTCHIN, E. (1961). Canine ovarian neoplasms. *Res. Vet. Sci.*, **2**, 133.

ENGLE, E.T. (1964). Tubular adenomas and testis-like tubules of the ovaries of aged rats. *Cancer Res.*, **6**, 578.

FOX, H. & LANGLEY, F.A. (1976). *Tumours of the ovary*. Heinnemann: London.

KNOWLES, J.F. (1982). The effect of X-radiation given after neonatal administration of ethyl nitrosourea on incidence of induced nervous system tumours. *Neuropath. Appl. Neurobiol.*, **7**, 265.

KNOWLES, J.F. (1983). Neonatal ethyl nitrosourea and X-radiation: carcinogenic effects in rats kept for their complete lifespan. *J. Natl Canc. Inst.*, (in press).

MURPHY, E.D. (1966). Characteristic Tumours. In *Biology of the Laboratory Mouse*, pp. 521–562. Ed. E.L. Green. McGraw-Hill: New York.

SCHMAHL, W. & KRIEGEL, H. (1978). Oncogenic properties of transplacentally acting ethyl nitrosourea in NMR1—mice after antecedent X-irradiation. Z. Krebsforsch., **91**, 69.

SYMINGTON, T. & CAMERON, K.M. (176). Endocrine and genetic lesions. In *Pathology of the testes*, pp. 259. (Ed. R.C.B. Pugh). Blackwell: Oxford.

ULLRICH, R.L. & STORER, J.B. (1979). Influence of $\gamma$ irradiation on the development of neoplastic disease in mice. II. Solid tumours. *Radiat. Res.*, **80**, 317.

VESSELINOVITCH, S.D., RAO, K.V.N., MIHAILOVITCH, N., RICE, J. & LOMBARD, S. (1974). Development of broad spectrum of tumours by ethyl nitrosourea in mice and the modifying role of sex, age and strain. *Cancer Res.*, **34**, 2530.